# A Comparative Evaluation of Measurement-Based Psychiatric Care Delivered via Specialized Telemental Health Platform Versus Treatment As Usual: A Retrospective Analysis

**DOI:** 10.7759/cureus.21219

**Published:** 2022-01-13

**Authors:** Sara Chokshi, Yalini Senathirajah, Vandana Yadav, Mimi Winsberg, Erin O’Callaghan, Scott Sullivan, Abhishek Verma, Stan Kachnowski

**Affiliations:** 1 Research, Healthcare Information Technology Lab (HITLAB), New York, USA; 2 Department of Biomedical Informatics, University of Pittsburgh School of Medicine, Pittsburgh, USA; 3 Research and Strategy, Healthcare Information Technology Lab (HITLAB), New York, USA; 4 Psychiatry, Brightside, San Francisco, USA; 5 Psychology, Brightside, San Francisco, USA; 6 Analytics, Brightside, San Francisco, USA; 7 Department of Biomedical Informatics, University of Pittsburgh, Pittsburgh, USA; 8 Columbia Business School, Columbia University, New York, USA; 9 Department of Management Studies, Indian Institute of Technology - New Delhi, New Delhi, IND

**Keywords:** remission induction, treatment outcome, depression, psychiatry, telemedicine

## Abstract

Background and objective

A significant proportion of the adult population in the United States (US) live with some form of mental illness. The more prevalent conditions of depression and anxiety are typically managed in primary care settings rather than specialty care. The aim of this study was to determine the efficacy of a novel, measurement-driven psychiatric treatment platform delivered via an online telemental health platform as compared to treatment as usual (TAU).

Methods

The TAU dataset and the telemental health platform (Brightside) dataset were constructed based on the total populations of adult patients receiving care for depression from January 2018 through December 2020 (November 2018 through March 2021 for the Brightside group). Patients in both groups had a primary mental health diagnosis of depression and the presence of a positive screen for depression as measured by the Patient Health Questionnaire-9 (PHQ-9) upon initiation of treatment. HITLAB, an independent digital health verification and testing lab, conducted comparative analyses of the two groups using the Chi-square test of independence.

Results

Close to 80% of telemental health platform patients experienced a reduction of 5 or more points from their baseline PHQ-9 score as compared to 52% of TAU patients. The mean reduction in PHQ-9 score was slightly higher in the Brightside group (-11.5) versus the TAU group (-10.1). Chi-square tests of independence [x2 (1, n=6281) = 256.75, p≤0.001] for meaningful reduction and for remission [x2 (1, n=6281) = 105.50 p≤0.001] were highly significant.

Conclusion

The telemental health platform patients performed significantly better than those under psychiatric TAU in terms of reduction in symptoms of depression in adults.

## Introduction

Close to one billion people worldwide and one in every five adults in the United States (US) live with some form of mental illness. From anxiety and addiction to depression and schizophrenia, mental health disorders vary in type and severity. Anxiety and depression are the two most common mental health conditions and contribute significantly to the burden of illness on the healthcare systems worldwide; care costs and reduction in productivity cost the global economy close to $1 trillion dollars annually and that amount is estimated to reach $6 trillion by 2030 [1-3].

The high prevalence of mental health disorders [like depression, anxiety, obsessive-compulsive disorder (OCD), posttraumatic stress disorder (PTSD)], which has only increased since the onset of the coronavirus disease 2019 (COVID-19) pandemic [4,5], has further amplified the long-standing marked shortage of mental healthcare providers, particularly psychiatrists in the US [6]. In areas where access to quality healthcare is traditionally lacking, psychiatry services are especially difficult to access [7], and psychiatric care is often provided in primary care settings by primary care physicians (PCPs) [8]. PCPs are generalists, with relatively little training in treating behavioral health disorders or on the wide array of psychiatric medications available to treat depression and anxiety disorders [9-13]. As psychiatric care represents a small slice of the multifocal primary care visit, there is less prioritization of mental health services, less time is devoted to rigorously measuring and monitoring mental health symptoms [14], and medication selection by PCPs is often found to be less varied and doses less frequently modified [15-16].

Despite the clearly proven benefits of measurement-based care, less than 20% of psychiatrists use it in their practice, emphasizing the need for better solutions to facilitate more widespread adoption of measurement-based care [17]. The recent technological advancements in medicine have led to the development of innovative interventions such as measurement feedback system technology, medical algorithms to help clinicians adhere to evidence-based guidelines, incorporation of measurement of mental health outcomes in electronic medical records (EHRs) [18], and a plethora of smartphone applications, to improve outcomes in mental healthcare [19].

Academic organizations have also developed simple measurement-based care platforms for electronically capturing patient-reported outcomes [20]. More intricate and costly technology with a measurement-feedback system linked to guideline recommendations and decision support is also available [19,21]. For organizations not using EHRs, there are point-of-care web-based, measurement-based care solutions like Owl Insights (Owl, Portland, OR) and VitalSigns6 that facilitate screening patients for mental health conditions, tracking their progress with patient-reported outcomes, and providing guidance for evidence-based treatment [22,23].

Finally, the emergence of online and mobile applications as powerful health technology tools, particularly highlighted during the COVID-19 pandemic, has made digital interventions increasingly important for mental healthcare [24]. With the surging interest in developing mental health applications (MHApps), various studies have been conducted to assess the efficacy of MHApps in enabling the treatment of psychiatric illnesses, most commonly depressive and anxiety disorders. Meta-analyses of these studies have demonstrated that patients using smartphone apps show a greater improvement in symptoms vs. controls, with a greater effect when compared with inactive than active control conditions, encouraging the integration of MHApps into the treatment of important and common psychiatric illnesses [25,26]. Many of these tools empower patients by enabling them to track their progress and become active participants in their healthcare journeys [27]. Moreover, many apps serve as useful methods for monitoring and facilitating early identification of risk and thus help mitigate negative psychiatric outcomes [28].

With the aim of addressing the shortage of psychiatric care and the lack of evidence-based and measurement-based approaches to medication prescription, Brightside offers a telemental health platform with built-in clinical decision support. This practice management and communication platform delivers patient care guidelines and suggests precision-prescribing to treating clinicians (a mixture of primary care and psychiatric providers) based on symptom cluster presentation. Additionally, the Brightside platform uses a measurement-based approach to tracking outcomes. Via the platform, clinicians prescribe from a wide array of psychotropic medications and use remote monitoring tools to evaluate patients both asynchronously and synchronously, thereby enabling fine-tuned and frequent treatment adjustments.

This study examines the effectiveness of a telemental health platform compared to treatment as usual (TAU) in significantly reducing symptoms of depression in adults. The telemental health platform approach is predicated on the hypothesis that algorithmic clinical decision support, plus frictionless communication between patient and prescriber, along with more frequent measurement-based assessments and medication adjustment, yields better outcomes for patients. While Brightside offers psychiatric services alone as well as in combination with therapy, the focus of this study is on the platform’s core offering of psychiatric care and medication delivery.

The objective of this retrospective cohort study is to identify and analyze the differences between depression TAU and novel, measurement-driven psychiatric treatment delivered through an online telemental health platform.

## Materials and methods

This study retrospectively compared datasets from two groups. One group received standard of care for depression in a large Midwestern health system (TAU). Much of the psychiatric treatment in the TAU setting happens in the context of wider health concerns [6]; therefore, patients in the TAU group had varied reasons for their visits, courses of treatment, and types of encounters. The TAU group provides a comparison for the intervention group (“platform”) in which patients were users of the Brightside platform.

**Figure 1:**
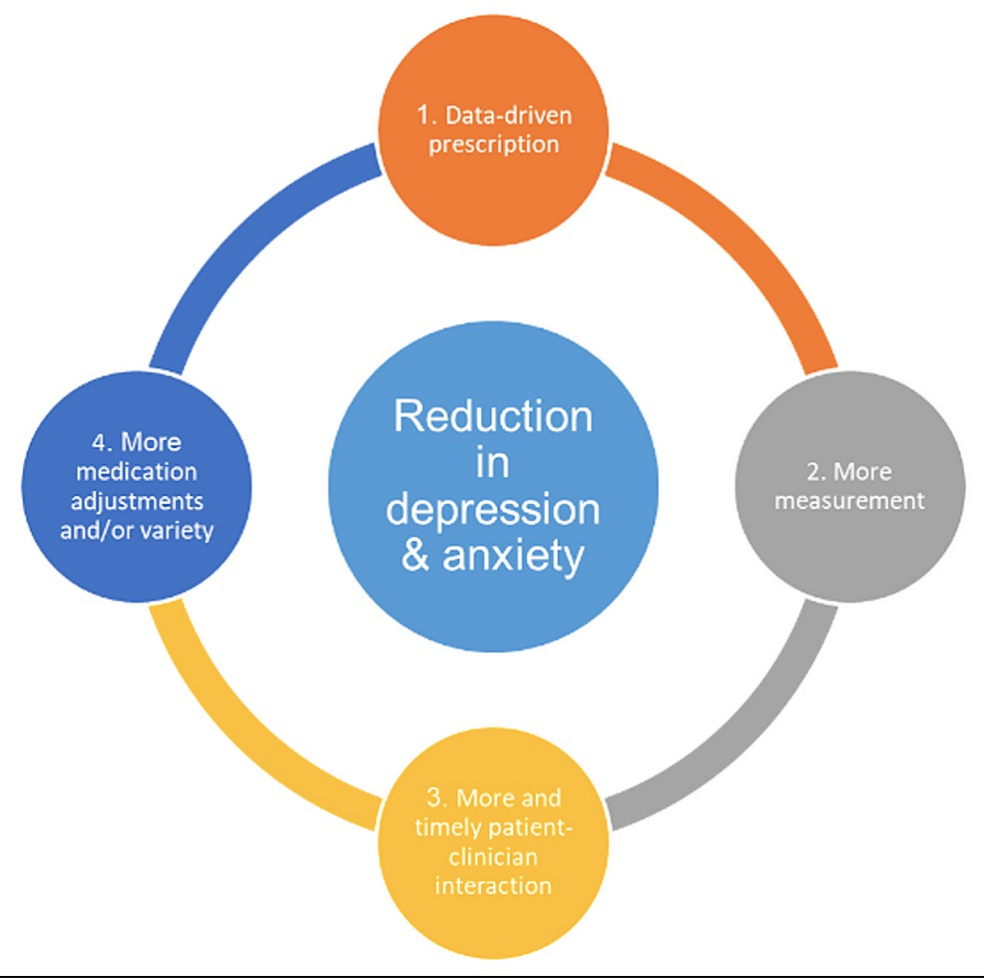
Telemental health platform’s model of care

Patients enrolled in the platform group completed a digital intake that included a clinically validated questionnaire, Patient Health Questionnaire-9 (PHQ-9), in addition to questions that assessed the clinical data points in the patients’ current presentation and history, such as sleep patterns, family history, and prior medication trials. Based on the symptom cluster analysis of these data points, the platform delivered patient care guidelines and suggested real-time precision-prescribing to the treating provider. The platform also used a measurement-based approach to track outcomes and alert providers in real-time when patients failed to improve, worsened, or experienced worrisome symptoms such as suicidal ideation. Platform clinicians, who were predominantly PCPs, communicated with patients both asynchronously via messaging and synchronously via video sessions (Figure 1).

The TAU dataset and the platform dataset were constructed from the total populations of patients receiving care for depression from January 2018 through December 2020 (November 2018 through March 2021 for the Brightside group). Patients in both groups were selected for inclusion based on age (adults, 18-49 years), a primary mental health diagnosis of depression [determined by select International Classification of Diseases (ICD)-9 or 10 codes], and the presence of a positive screen (10 points or higher) for depression as measured by the PHQ-9 upon initiation of treatment. Additionally, to qualify for inclusion, participants in both groups must have had at least one additional PHQ-9-based assessment occurring at 8-16 weeks (endline), and a prescription on record of at least one psychotropic medication.

In both groups, patients were treated predominantly by PCPs; however, the proportion of patient encounters involving PCPs in the platform group was much higher (96%) than in the TAU group where slightly more than half (53%) of encounters involved a PCP versus a behavioral health specialist (47%).

The PHQ-9 is a self-reported measure of major depressive disorder (MDD) symptoms [29,30]. It is a brief measure (nine questions) of depression severity. Respondents rate each item on a 4-point Likert scale (0-3) with total scores ranging from 0 to 27 (higher scores reflect greater depression severity). The PHQ-9 demonstrates strong reliability and validity with 88% sensitivity and 88% specificity for MDD. Participants were administered baseline screening surveys (before the start of the treatment), and these same surveys were administered every two weeks throughout their treatment period. All subjects in both groups received a prescription for at least one psychiatric medication during the study period. Patients were excluded if they had certain other mental health diagnoses (psychosis, schizophrenia, or bipolar 1 disorder) or specific comorbid chronic health conditions that require active lab monitoring (e.g., chronic kidney or liver disease).

To compile the TAU group dataset, anonymized electronic health record (EHR) data was obtained from the health system research data service, after Institutional Review Board review, as .csv files. Jupyter Notebooks, an open-source web application, was used to perform the analysis. Pandas, NumPy, and Matplotlib were the primary Python packages used for data extraction, data cleaning, data processing, and data visualization. The data types included encounter dates and types, conditions with ICD-10 diagnostic codes, PHQ-9 details and dates, medication orders and refills, and demographic data for patients seen in the primary care department. The basic demographic data based on the dataset of those with treatment between 8-16 weeks with two or more PHQ-9 administrations, with initial scores above 10, yielded 710 patients. The demographic information for participants in both the platform and TAU groups is presented in Table 1.

An independent digital health verification and testing lab coordinated dataset construction and conducted the comparative analysis for the two groups. For efficiency and to maximize patient privacy, authors from each institution created and performed analyses on datasets for their respective groups (TAU, platform). Each group, after obtaining the appropriate Institutional Review Board approval, summarized and delivered datasets to the lab team for statistical comparison of the two groups as described below. 

Statistical analysis

Descriptive analyses were performed for each of the TAU and platform datasets to ascertain the proportion of participants experiencing a minimal clinically important difference (MCID) in PHQ-9 scores from initiation to endline in each group. Similarly, the proportion of participants experiencing remission (score of <10 on PHQ-9 at endline) in each group was also examined. While a score of 10 or higher served as the threshold for a disorder, a difference of 5 or more points on the PHQ-9 was defined as an MCID for this study [30].

To compare the proportions of patients experiencing an MCID in their PHQ-9 score and those achieving remission in the two groups, a Chi-square test of independence was performed. Results of these tests are presented in Tables 2, 3 and described in detail in the following section.

## Results

Group characteristics

Overall, the TAU and platform groups were roughly similar in terms of gender and age but not racial distribution (Table 1). Both groups had a skewed female distribution (approximately 70% female) and had the highest proportion of patients in the 25-34-year-old age bracket. Approximately 80% of patients in both groups identified as white, while the TAU group had more (15% vs. 3.7%) patients who identified as Black. Hispanic ethnicity was not identified in the TAU dataset; however, 8% of the platform participants identified as Hispanic. The Brightside participants spanned the socioeconomic spectrum. Platform participants were slightly more symptomatic with a mean baseline PHQ-9 score of 18.0 vs. that of 16.6 in the TAU group.

**Table 1:** Descriptive comparison of platform and TAU groups *Race/ethnicity recorded for 698 out of the 710 patients in the TAU group PHQ-9: Patient Health Questionnaire-9; TAU: treatment as usual

Characteristics	Categories	TAU	Platform
		N=710	N=5571
Gender			
	Female	72.6%	71.3%
	Male	27.4%	28.7%
Age (years)			
	18-24	27.9%	23.0%
	25-34	42.0%	54.8%
	35-44	29.2%	19.3%
	45-49	04.2%	03.0%
Race		N=698*	N=5571
	White	81.0%	78.6%
	Black	15.5%	03.7%
	Asian	02.1%	03.6%
	Hispanic	Unavailable	08.1%
	Native American	00.3%	00.4%
	Pacific Islander	00.0%	00.4%
	Other	Unavailable	05.2%
Baseline PHQ-9 score		16.6	18.0

Platform patients in the study received over 1,000 medication/dose combinations, and more than half of these patients had at least one medication adjustment within the study period. Platform patients experienced an average of 13.3 clinical touchpoints throughout the study period, which included synchronous video consults, asynchronous provider messages sent and received, case reviews, and check-in surveys. Additionally, platform patients averaged 3.7 days from the time of enrollment to first appointment (50.8% of patients were treated within 48 hours of enrollment, 68.7% within 72 hours, and 78.9% within 96 hours).

The proportion of patients with a minimal clinically important reduction

**Figure 2:**
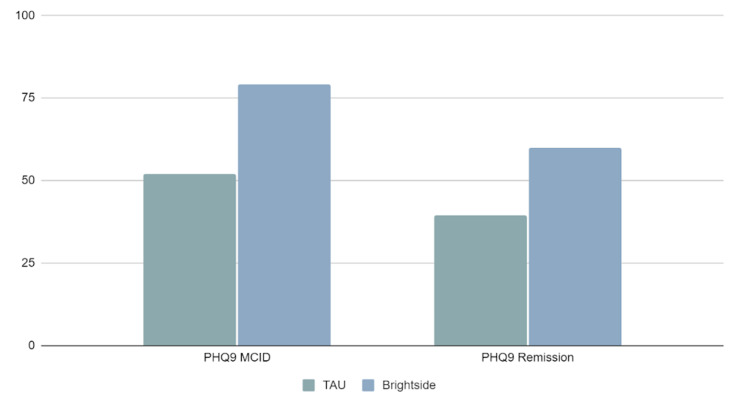
Percentage of patients reporting PHQ-9 scores with a minimal clinically important difference (MCID) (5+ reduction) and remission at endline PHQ-9: Patient Health Questionnaire-9; TAU: treatment as usual

Proportions of patients experiencing MCID are featured in Figure 2, along with the proportion of patients who started out as depressed or anxious and then measured below 10 (remission) on endline administration of the PHQ-9. Patients in the platform group were significantly more likely to experience both MCID as well as remission from initiation to endline. Chi-square tests of independence [x2 (1, n=6281) = 256.75, p<0.001] for MCID and for remission [x2 (1, n=6281) = 105.50 p<0.001] are shown in Tables 2, 3.

Close to 80% of platform patients experienced a 5 or more-point reduction in their baseline PHQ-9 score as compared to 52% of TAU patients. The mean reduction in PHQ-9 score was slightly higher in the Brightside group (-11.5) vs. the TAU group (-10.1).

Similar results were found for patients who achieved remission (an endline score of <10 points). Almost 40% (39.6) of TAU patients achieved remission by the end of the study period, while close to 60% (59.8) of platform patients achieved remission; that proportion rises to more than 73% when PHQ-9 scores are considered.

**Table 2:** Percentage of patients reporting a minimal clinically important difference (MCID) and remission at endline PHQ-9: Patient Health Questionnaire-9; TAU: treatment as usual

	TAU (n=710)		Brightside (n=5571)	
	%	N	%	N
Percentage of patients achieving MCID as per PHQ-9 (5+ reduction)	52.0	369	79.2	4412
Percentage of patients achieving remission (score <10) as per PHQ-9	39.6	281	59.8	3332

**Table 3:** Differences between platform and TAU groups in the proportion of patients experiencing PHQ-9 MCID (reduction) and remission PHQ-9: Patient Health Questionnaire-9; MCID: minimal clinically important difference; TAU: treatment as usual

	TAU	Platform
MCID		
Sample proportion	0.52	0.79
95% CI	0.51-0.53	0.77-0.82
Z-value	13.7	
P-value	0	
Remission		
Sample proportion	0.40	0.60
95% CI	0.39-0.41	0.57-0.63
Z-value	10.3	
P-value	0	

## Discussion

The objective of this study was to ascertain how a novel, platform-based psychiatry service compares to TAU in the treatment of patients with depression. To our knowledge, this is the first known study to compare patient outcomes between a novel, platform-based psychiatry service to TAU. Given the shortage of psychiatric service providers, primary care has become a front-line option for patients who experience mental health disorders. PCPs, however, must be attuned to a variety of health concerns and chronic conditions and therefore have far less time and energy to devote to the often nuanced needs and treatment options for patients with depression. Telemental health platform-delivered psychiatric treatment offers the convenience of remote service, as well as asynchronous touchpoints, frequent remote patient monitoring, and measurement-based care with validated tools.

Results from this study demonstrated that a statistically greater number of patients in the platform group showed improvements in depression symptoms from baseline to endline treatment when compared to the group of patients who received psychiatric TAU. More specifically, 79% of patients in the platform group showed an MCID in symptoms of depression, as measured by the PHQ-9, whereas 52% of the TAU group demonstrated MCID in symptoms of depression. Although platform patients were somewhat more symptomatic on average at treatment initiation, a higher proportion of these patients were able to achieve remission when compared to those treated with TAU; 40% of patients treated with TAU achieved remission, whereas 60% of platform patients achieved remission.

The results of this study must be considered in light of its limitations. The study was retrospective in design, and as such, there was an inherent selection bias in that all patients treated in both groups chose to pursue psychiatric treatment for their depression. While all patients in the study from both groups were prescribed at least one psychotropic medication, patients in both groups may or may not have received psychotherapy in addition to psychiatric medication, which may have had an impact on changes in PHQ-9 scores. Therefore, conclusions about whether or not the greater reductions in depression symptoms and remission rates seen in the platform group were due to the unique offerings of the platform, such as the frequent assessments paired with the prescribing clinical decision support for medication selection, cannot be made. Furthermore, the study was restricted to the data available for retrospective analysis, and the restrictions on the use of patient data only allowed for analysis in aggregate.

Based on the findings from this retrospective analysis, a randomized controlled trial is required in order to further evaluate the impact of psychiatric intervention provided via telemental health platform vs. TAU. Future research should also address how key elements of the telemental health platform (e.g., more frequent measurement, opportunity for asynchronous touchpoints) impact both the patient and clinician experience and satisfaction in relation to usual care. Given the surge in need for mental health and psychiatric services in the wake of the COVID-19 pandemic and what continues to be a deficit in the number of specialty care providers, psychiatric care delivered via a telemental health platform like Brightside offers an important option that can provide psychiatric care to those who may not otherwise have access to it.

In addition to limitations related to the retrospective design of the current study, there were several other limitations of note. While the gender composition of both groups was close to identical, both groups skewed heavily toward females and whites. Additionally, the platform group contained very few Black patients overall and fewer in comparison to the TAU group. Future research should examine if similar results as in this study can be achieved in groups with a greater proportion of non-white patients. 

Finally, while a strength of the telemental health platform lies in the access to complete and comprehensive patient data for these kinds of analyses, generating a comparable dataset from EHR data can be challenging as was the case in this study, making it difficult to compare medication and encounter data across the platform and TAU patient samples. Future research should seek to identify ways by which to better compare telemental health patients’ medication practices and clinical touchpoints with those experienced in psychiatric treatment in typical primary care settings.

## Conclusions

The telemental health platform incorporating clinical decision support and facilitating measurement-based care led to greater reductions in depressive symptoms and greater remission rates compared to psychiatric TAU. Since this was a retrospective cohort study, conclusions about the efficacy of the platform could not be drawn. More research, such as a randomized control study, is needed to understand the role that specific functions facilitated by telemental health platforms, such as frequent touchpoints and regular measurement-based assessments, play in yielding better outcomes for patients with depression.
